# The Role of Vitamin D in Gastrointestinal Homeostasis and Gut Inflammation

**DOI:** 10.3390/ijms26073020

**Published:** 2025-03-26

**Authors:** Varun Vemulapalli, Anusha Shirwaikar Thomas

**Affiliations:** 1 Department of Internal Medicine, The University of Texas Health Science Center at Houston, Houston, TX 77030, USA; 2 Department of Gastroenterology, Hepatology, and Nutrition, The University of Texas MD Anderson Cancer Center, Houston, TX 77030, USA

**Keywords:** gastrointestinal homeostasis, Vitamin D, gastrointestinal, gut inflammation, microbiota, gut microbiome

## Abstract

Gastrointestinal homeostasis describes a delicate state of equilibrium in which various systems cooperate to maintain digestive health, support microbial activity, and regulate immune responses. There is growing evidence that Vitamin D is one of the many factors that influences gastrointestinal homeostasis through its effects on gut barrier integrity, regulating microbial diversity and modulating immune responses. Given these effects of Vitamin D, there may be potential for it as both a preventative and a therapeutic intervention for a variety of conditions, but especially for inflammatory conditions of the gastrointestinal tract. This article will summarize the role of Vitamin D in a state of equilibrium, as well as its role in a pro-inflammatory state in the gastrointestinal tract.

## 1. Introduction

Intestinal homeostasis is a state of equilibrium that involves a complex interplay of multiple systems that work together to maintain digestive health, immune regulation, and microbial function. This homeostasis is crucial for overall health and well-being, as the intestines are not only responsible for nutrient absorption, but also play a central role in immune defense, microbial balance, and even the communication between the gut and the brain [1]. There are many factors affecting gastrointestinal (GI) homeostasis, and there is growing evidence that Vitamin D is one such factor [2]. While the conventional role of Vitamin D is to maintain skeletal homeostasis, it also plays a critical role in maintaining GI homeostasis by maintaining gut barrier integrity, microbiome composition, immune regulation, and inflammation control [3]. These effects contribute to overall gut health, digestive efficiency, and disease prevention. This article will summarize the impact Vitamin D has on gut homeostasis and inflammation through its effects on the intestinal epithelium, the microbial diversity of the gut, and the immune system.

## 2. Vitamin D: Production, Metabolism, and Mechanisms of Action

**Figure 1:**
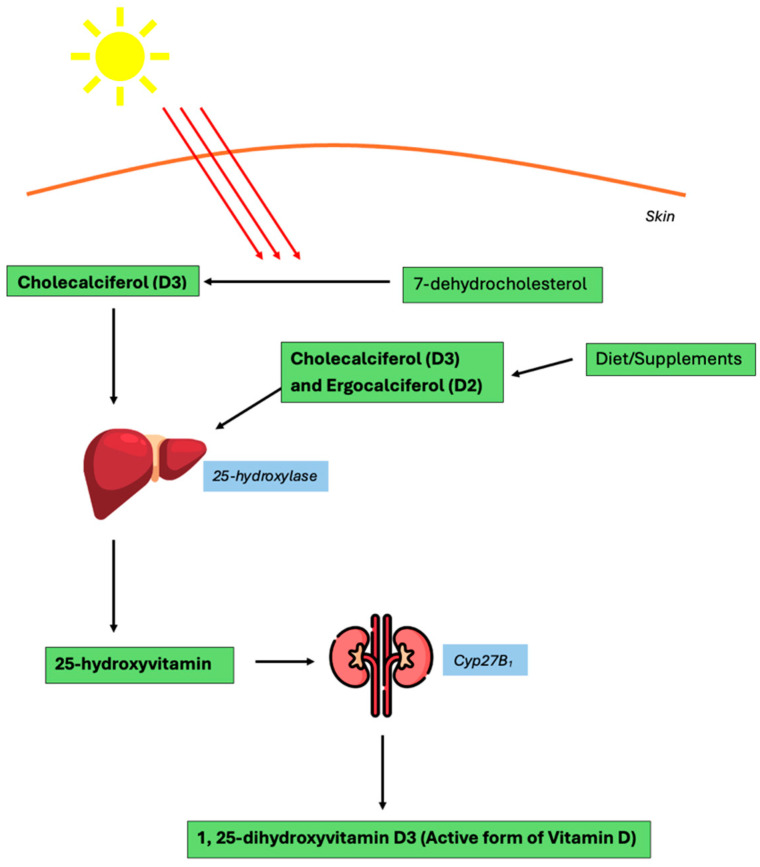
Vitamin D biosynthesis pathway [2,4].

Vitamin D is a fat-soluble vitamin that can be obtained through nutrition or synthesized by the body after sunlight exposure. Through nutrition, Vitamin D is available in the form of cholecalciferol (D3) or ergocalciferol (D2), both of which are inactive forms of Vitamin D. In contrast, sunlight exposure induces Vitamin D synthesis by catalyzing the conversion of 7-dehydrocholesterol to cholecalciferol [4]. For Vitamin D2 and D3 to be converted to the biologically active form of Vitamin D, they must first be hydroxylated in the liver, specifically by the enzyme 25-hydroxylase, to 25-hydroxyvitamin D [25(OH)D]. Vitamin D primarily circulates in this inactive form and is stored in the liver and adipocytes [2]. Then, 25(OH)D must be further hydroxylated in the kidney by the enzyme Cyp27B_1_ to the biologically active form of Vitamin D, 1,25-dihydroxyvitamin D3 [1,25(OH)_2_D_3_] (Figure 1) [2,4]. The active form of Vitamin D is responsible for initiating the processes that ultimately regulate GI homeostasis. In addition to being dependent on Cyp27B_1_, Vitamin D levels are also affected by serum calcium and parathyroid hormone (PTH) levels. Low serum calcium levels lead to inadequate activation of calcium sensing receptors, which triggers PTH secretion [2]. This signaling cascade facilitates intestinal calcium absorption, renal tubular reabsorption, and bone demineralization to increase serum Vitamin D levels.

The biological activity of Vitamin D is mediated by the Vitamin D receptor (VDR), a ligand-activated transcription factor belonging to the nuclear receptor superfamily [4]. VDRs are widely distributed throughout the small and large intestines, as well as in various immune cells, including monocytes, macrophages, dendritic cells, and activated B and T lymphocytes [3]. Upon binding to Vitamin D, VDR initiates intracellular signaling pathways that influence multiple physiological functions. In the gut, VDR is highly expressed in healthy intestinal epithelial cells (IECs), particularly within the IEC crypts [4]. Emerging evidence suggests that Vitamin D/VDR signaling plays a crucial role in immune modulation. While the precise mechanisms remain unclear, the widespread expression of VDR in immune cells supports its role in immune regulation.

**Figure 2:**
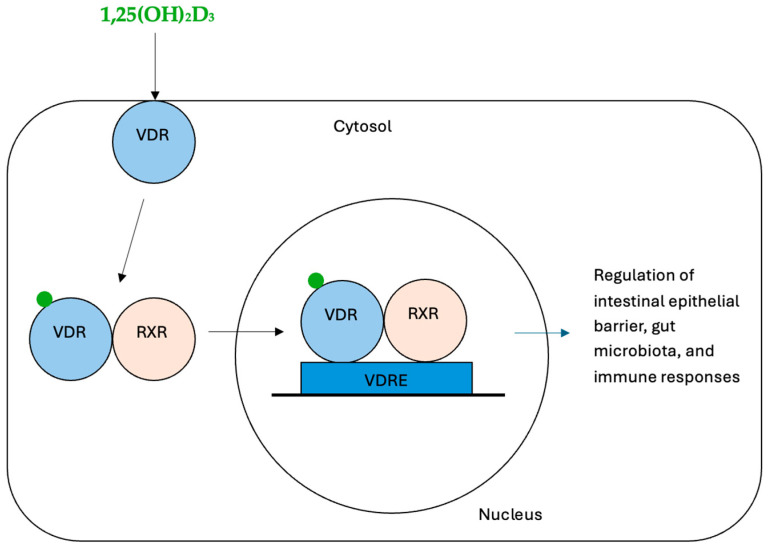
Vitamin D and VDR/RXR signaling [5,6,7,8,9].

After Vitamin D binds to VDR, one way in which it exerts its biological effects is by forming a heterodimer with the Retinoid X Receptor (RXR), another nuclear receptor involved in the regulation of various key physiological process and inflammatory pathways [5]. The VDR–RXR complex subsequently translocates to the nucleus, where it binds to Vitamin D Response Elements (VDREs) in the promoter regions of target genes. By recruiting coactivators or corepressors, this complex modulates gene expression to exert its regulatory functions [6] (Figure 2).

The interaction between VDR and RXR is dynamic and functionally significant. RXR is not limited to partnering with VDR; it can also form homodimers or heterodimers with other nuclear receptors such as the liver X receptor (LXR), the peroxisome proliferator-activated receptor (PPAR), and the retinoic acid receptor (RAR). These alternative RXR pairings regulate critical pathways related to lipid metabolism, inflammatory responses, and homeostatic processes [7]. However, when RXR dimerizes with VDR, it prioritizes VDR-mediated transcriptional regulation, significantly impacting the expression of genes involved in gut homeostasis [6]. With its increased affinity to the DNA VDREs to facilitate gene expression, the VDR–RXR heterodimer becomes a significant factor in regulating intestinal epithelial barrier integrity, modulating microbiota composition, and orchestrating immune responses [8]. The following sections will explore these functions, and their relationship with Vitamin D, in greater detail.

## 3. The Gut Mucosa and Vitamin D 

### 3.1. Mucus Layer

The GI tract is a complex, layered structure with each layer responsible for varying functions, many of which are affected by Vitamin D. The innermost layer, the mucosa, is composed of the epithelium, the lamina propria, and the muscularis mucosa [3,10]. Resting on top of the epithelium is a mucus layer composed of mucin glycoproteins that aggregate to maintain a protective barrier between the intestinal lumen and the host epithelial cells [3]. The mucus layer can be subdivided into two layers: a loosely adherent outer layer and a dense, firmly adherent inner layer. The outer layer harbors bacteria and serves as a site for bacterial proteolytic activity, while the inner layer is less susceptible to bacterial penetration due to its thickness [3,11]. Goblet cells, found along the intestinal epithelium, are responsible for maintaining the mucus layers to protect against pathogens and immunogenic products. As the outer layer of mucus is degraded by proteolytic bacterial activity, it is replaced by the pre-existing inner mucus layer, while the goblet cells work to replace the inner mucus layer [11].

Regarding the role of Vitamin D in this process, animal models have demonstrated thinner mucus layers in mice that are deficient in the Cyp27B_1_ enzyme. In these studies, mice were found to have abnormal goblet cell storage and mucus production, both of which were resolved with the correction of serum calcium abnormalities [3,12]. This strongly points to the indirect effect of Vitamin D in mucus production in which the regulation of serum calcium levels by Vitamin D is essential for the adequate function of goblet cells. In the setting of a compromised mucus layer, whether due to dysbiosis or an inflammatory state, Vitamin D supplementation may be considered to increase mucus secretion and strengthen the first line of intestinal pathogen defense (Table 1).

### 3.2. Epithelium

The epithelium lies beneath the mucus layer and serves as the second barrier for invasive pathogens. Epithelial cells are securely connected by tight junctions (TJs) and adherens junctions (AJs), which are composed of proteins such as occludins, claudins, and E-cadherins [3]. Animal studies demonstrate that Vitamin D/VDR signaling specifically enhances claudin-2 promoter activity and that there was a decrease in claudin-2 activity in mice that were VDR deficient [19]. Therefore, Vitamin D is essential to this pathway, as it indirectly upregulates the expression of the junctional proteins that comprise TJs and AJs to strengthen the mucosal barrier. Loss of these junctional components can compromise the gut epithelial integrity and increase permeability to pathogenic and immunogenic products [3,20]. This process of junctional protein loss has been hypothesized to be a contributing or precipitating factor in inflammatory bowel disorders [21]. In fact, in vitro studies have shown that Vitamin D supplementation can upregulate the formation of TJs and reduce gut permeability, thereby reducing inflammation [13]. These findings support maintaining adequate levels of Vitamin D, which may stabilize the mucosal barrier and thereby serve as a protective factor in immune-mediation or inflammatory bowel conditions.

The epithelial layer is where VDR is most prevalent, and the integrity of this layer is regulated by Vitamin D/VDR signaling [14]. Because colonic mucosal permeability predisposes one to developing inflammation in the intestines, it stands to reason that VDR dysregulation is implicated in IBD. Studies with mice in the dextran sodium sulfate-induced colitis model showed an increase in mucosal permeability in VDR-deficient mice through the observation of reduced transepithelial electrical resistance (TER) levels [14,15]. TER is a measurement of the passage of ions and solutes over the epithelium used to assess the integrity of the intestinal barrier [22]. With this measurement, a higher value indicates a tighter barrier. In these studies, VDR-deficient mice also developed more severe colitis and had higher mortality rates when compared to VDR-sufficient mice [16].

IEC apoptosis is a significant contributor to disruption and damage of the epithelial barrier [17]. The process of IEC apoptosis and disruption of the intestinal epithelium is independent of TJs. The primary mode in which this occurs is through the activation of p53 upregulated modulator of apoptosis (PUMA), a pro-apoptotic protein that is targeted by tumor suppressor protein p53 to induce apoptosis in various cell types [23]. When p53 is activated, it stimulates and directs PUMA to bind to other pro-apoptotic proteins, Bax and Bak, to trigger mitochondrial dysfunction. The subsequent release of cytochrome c leads to caspase activation and eventual cell death [17,24]. PUMA, which is mediated by tumor necrosis factor alpha (TNF-α) and interferon gamma (IFN-γ), has been proven to be a significant factor in the pathogenesis of IBD, as tissue samples from patients in several studies have revealed correlations between IBD disease activity and elevated levels of PUMA [24,25]. TNF-α is induced by the transcription factor nuclear factor-kappa B (NF-κB), and we know from prior studies that Vitamin D inhibits the activation of NF-κB [26]. Vitamin D is integral in inhibiting this process, as it induces VDR signaling that prevents the activation of NF-κB through inhibition of IKK kinase, an enzyme implicated in colitis development and PUMA activity [17]. This signaling cascade establishes Vitamin D/VDR signaling as an inhibitor of PUMA through inhibition of TNF-α induction. This is supported by the observation that in trials of mice with significant IEC apoptosis, the introduction of the hVDR transgene, which is responsible for VDR synthesis, was found to down-regulate the PUMA [14]. This process by which Vitamin D/VDR signaling suppresses IEC apoptosis to maintain the integrity of the epithelial barrier and decrease mucosal inflammation suggests a potential role for Vitamin D as a therapeutic intervention in IBD (Table 1).

Several studies have established that there is a strong relationship between Vitamin D deficiency and IBD [27]. While Vitamin D deficiency is a common finding in patients with IBD and is associated with increased disease activity, it is unclear whether it is a predisposing factor in IBD development or a consequence of poor disease control [14]. In a study between two cohorts of IBD patients, one with normal Vitamin D levels that received Vitamin D supplementation and one with low Vitamin D levels that did not, a common finding was a reduction in epithelial VDR expression [17]. This shared characteristic between the two cohorts, despite varying Vitamin D levels, indicated that decreased VDR expression in intestinal epithelial cells is independent of Vitamin D levels and is likely an inherent characteristic of IBD. Although the mechanism of downregulation of VDR in IBD is unclear, various studies have supported the relationship between an inflammatory state and reduced VDR expression [28,29].

Similar to the relationship between Vitamin D and IBD, there is growing evidence that there is also an association between Vitamin D and immune mediated colitis (IMC). Immune mediated colitis is a complication of immune checkpoint inhibitor (ICI) therapy that is used in the treatment of various malignancies [30]. ICIs work by blocking the immune checkpoint pathways that normally halt immune responses. ICIs also decrease the activity of T regulator (Treg) cells, which suppress immune responses through their production of anti-inflammatory cytokines [31]. Through these processes, ICIs allow for an enhanced immune response that has proven to be effective in attacking cancer cells. However, this enhanced immune response increases the risk of autoimmunity, the process by which the immune system attacks healthy tissue [32]. In the case of IMC, autoimmunity and lymphocytic infiltration of the intestinal epithelium leads to increased inflammation and IEC damage. As with other inflammatory disorders of the GI tract, there is evidence that the gut microbiome plays a role in the development of IMC, although the exact association is not clear. Because the phenotypical, histological, and serological characteristics of IMC are similar to those of IBD, one may speculate about the protective role of Vitamin D against IMC, although further studies are needed to clarify this role [33].

It should be acknowledged that while there is evidence to suggest that there is a positive relationship between Vitamin D and IBD, the full effects of Vitamin D supplementation on the disease course of individuals with IBD remain inconclusive. There are many studies that have observed Vitamin D supplementation to decrease symptomatology and disease activity in this population [34,35,36]. In fact, one review of several randomized control trials of patients with IBD receiving Vitamin D supplementation and placebo did show that there may be a decrease in clinical relapses of the disease after supplementation. However, it also stated that no clear conclusion could be made on whether this intervention resulted in significant clinical response or remission of IBD [27]. Furthermore, one study observed an increase in disease activity after Vitamin D supplementation in patients with ulcerative colitis (UC) [37]. While this increase was minimal, these results, which are inconsistent with other studies, highlight the potential limitations of Vitamin D supplementation in IBD. Ultimately, further studies are necessary to clarify the influence of Vitamin D on gut homeostasis, specifically in individuals with IBD.

### 3.3. Gut Microbiome

The intestinal microbiome is a dynamic and diverse environment in the GI system, made up of trillions of microorganisms, including bacteria, fungi, parasites, viruses, and archaea, that live in a symbiotic state to maintain equilibrium [38]. This environment can be disrupted and altered due to multiple factors, such as dietary changes, genetics, environment and lifestyle exposures, and age. In fact, the gut microbiome is home to three to five million genes, which is more than one hundred times more than the human genome [3]. While the gut microbiome is vital in the function of the gastrointestinal tract for food breakdown and nutrient absorption, the genetic diversity of the gut microbiome also allows it to have a strong influence in maintaining intestinal homeostasis. There is also growing evidence that disruption to the microbiome, or dysbiosis, plays a role in the severity and progression of inflammatory conditions such as inflammatory bowel disease (IBD) [39].

The bacteria in the intestinal microbiome are comprised of primarily anaerobic species (>90%), with aerobes and facultative aerobes making up only ~5% of the bacterial population [3]. These bacteria can be subdivided into two groups: autochthonous (resident) and allochthonous (transient) species. The former group consists of bacteria that are a mainstay in the GI system and contribute to the variety of functions of the gut microbiome. The latter group consists of pathogenic bacteria that are common in healthy individuals, but are transient due to their eradication by the host immune system. Bacteria in the intestinal microbiome are predominantly from four different bacterial phyla: *Firmicutes*, *Bacteroidetes*, *Actinobacteria*, and *Proteobacteria* [4]. In healthy individuals, members of the *Firmicutes* and *Bacteroidetes* phyla account for 90–95% of the bacterial population. This equilibrium of the gut microbiota can be affected by several factors, including Vitamin D. In fact, a study in which Vitamin D supplementation was given to women who were Vitamin D deficient, but otherwise healthy, showed significant increases in microbial diversity. Specifically, increases in the *Bacteroidetes*-to-*Firmicutes* ratio and of species in *Bifidobacterium* were observed [40].

Species of *Firmicutes* and *Bacteroidetes* phyla are key producers of butyrate, a short chain fatty acid (SCFA) that serves as a primary energy source for IECs (Table 2). IECs metabolize butyrate through B-oxidation, using it as fuel to support growth and function to maintain a healthy and functional epithelial layer [41]. Additionally, studies have shown that SCFAs, particularly butyrate, can enhance intestinal restitution and improve intestinal permeability even after injury to the epithelium [42]. Butyrate promotes the expression of genes encoding TJs and their components (claudins, occludins) through activation of various transcription factors. In doing so, butyrate has been found to maintain and/or increase TER in human colonic cells, as well as in various animal cells, even in a pro-inflammatory environment [43]. This serves to foster a healthy, non-permeable intestinal epithelial layer. Furthermore, butyrate supplementation has been found to increase VDR expression and suppress inflammation in colitis models [44]. These findings further highlight the role of butyrate in maintaining the intestinal barrier and also suggest a therapeutic role for butyrate in IEC restitution in inflammatory conditions such as IBD.

*Bifidobacterium* species, of the phylum *Actinobacteria*, are organisms also affected by Vitamin D that have been studied to potentially have protective effects in IBD and even in colorectal cancer (CRC). A study in which healthy individuals received Vitamin D supplementation for 12 weeks resulted in significant increases in the number of *Bifidobacterium* species [45]. In addition, *Bifidobacterium* supplementation in patients undergoing CRC surgeries was associated with a decrease in postoperative inflammation [46]. Given that individuals with CRC have been found to have decreased levels of SCFAs, the enhancement of SCFA production by *Bifidobacterium* species, as occurs with species of *Firmicutes* and *Bacteroidetes* phyla, is the likely explanation for this finding (Table 2) [47]. Other studies have also explored the relationship between *Bifidobacterium* species and SCFAs and support the idea that they promote SCFA production [48].

In IBD, Vitamin D supplementation has been shown to have significant effects on the microbial profile. The pathogenesis of IBD involves the proliferation of pathogenic microorganisms and the depletion of commensal organisms [49]. For example, species of *Proteobacteria*, such as *E. coli*, have been found to proliferate in states of dysbiosis, particularly in individuals with Crohn’s disease (CD) (Table 2) [12,50]. Certain pathogenic microorganisms have also been linked to specific complications of IBD, as the proliferation of *Proteobacteria* species has been associated with an increased incidence of structuring disease and need for surgery in individuals with CD [51]. The association between *Bacteroidetes* species and IBD has also been well studied, and levels of *Bacteroidetes* species in individuals with IBD, especially active IBD, were found to be notably decreased when compared to control groups [52]. In studies examining the role of Vitamin D in IBD, wild-type mice and VDR-deficient mice demonstrated significant differences in the composition of the respective microbiomes, suggesting a role for Vitamin D as a moderator of the gut microbiota. These studies also demonstrated improvement in colitis with Vitamin D supplementation in VDR-deficient mice [12]. Although the mechanism of this effect is unclear, changes in gut microbiota may have played a role. In fact, other studies have observed increases in Enterobacteriaceae after Vitamin D therapy, with subsequent decreases in inflammation in both UC and CD [4].

It is important to note that there are also conflicting studies that have observed Vitamin D supplementation to have minimal or inconclusive effects on the gut microbiota, primarily in healthy individuals. In a study of healthy individuals who received Vitamin D supplementation for eight weeks, biopsies of the entire GI tract after supplementation demonstrated increased microbial diversity primarily in the upper GI tract [53]. The limitation of the effects of Vitamin D to the upper GI tract in healthy individuals is a marked difference to its beneficial effects in those with IBD, a disease with frequent involvement of the lower GI tract [34,35,36]. Furthermore, one study of the effect of Vitamin D supplementation in deficient individuals with CD compared to healthy controls with Vitamin D deficiency demonstrated no significant changes in microbial diversity of stool samples after four weeks [54]. This trial found increased levels of *Parabacteroides*, a genus whose abundance is associated with decreased inflammation, in only the CD cohort after supplementation (Table 2). These data on the effects of Vitamin D supplementation in the gut microbial alteration of healthy individuals, while limited, suggest that Vitamin D is a modulator of the gut microbiome primarily in an inflammatory state and may have no such effect in healthy individuals. Since this contradicts other studies that have observed microbial changes in Vitamin D-deficient, but otherwise healthy, individuals after supplementation, further studies are warranted to clarify the exact nature of this relationship [40,45].

**Table 2:** Microorganism of the gut microbiome and the effect of Vitamin D on their prevalence and function [4,12,40,41,46,47,48,54,55].

	Phylum	Regulation by Vitamin D	Effects of Vitamin D Regulation
*Bacteroides* spp.	*Bacteroidetes*	Increased abundance	- Increases Bacteroides-to-Firmicutes ratio - Promotes SCFA production and anti-inflammatory responses
*Parabacteroides* spp.	*Bacteroidetes*	Increased abundance	- Promotes SCFA production and anti-inflammatory responses
*Bifidobacterium* spp.	*Actinobacteria*	Increased abundance	- Promotes SCFA production and anti-inflammatory responses - Associated with anti-inflammatory role in CRC
*Roseburia* spp.	*Firmicutes*	Increased abundance	- Promotes SCFA production and anti-inflammatory responses
*Ruminococcus gnavus*	*Firmicutes*	Decreased abundance	- Linked to gut dysbiosis and inflammation - Suppressed by Vitamin D
*Escherichia coli*	*Proteobacteria*	Decreased abundance	- Linked to gut dysbiosis and IBD - Suppressed by Vitamin D

Ultimately, while there are data to suggest that Vitamin D has both an indirect and direct relationship to the gut microbiota, the direct relationship is not yet definitive. In addition, established research does indicate that Vitamin D influences the gut microbiota in IBD, but further research is warranted to determine the exact nature of this relationship.

It should also be acknowledged that microbial differences after Vitamin D supplementation in various studies may be inconsistent due to differences in doses, treatment duration, and study demographics. These variables should be controlled before making definitive conclusions on the effects of Vitamin D on gut microbiota.

### 3.4. Immune System Regulation

There is increasing evidence that shows the crucial role of Vitamin D and VDR signaling in the etiopathogenesis of autoimmune diseases, such as multiple sclerosis (MS), rheumatoid arthritis (RA), diabetes mellitus (DM), and inflammatory bowel disease [56]. Studies have shown that low serum Vitamin D levels can predispose an individual to developing these conditions, and decreased in utero Vitamin D exposure has even been linked to increased risk for autoimmune DM and pancreatic conditions. In the GI tract, Vitamin D, through immune system modulation, plays a significant role in preventing pathogens and immunogenic products from affecting GI homeostasis [4].

### 3.5. Innate Immune System

The innate immune system is the first line of the defense of the body against infections. It is fast-acting and nonspecific, mounting a defense against a broad range of pathogens without needing prior exposure [57,58]. Vitamin D plays a significant role in regulating the innate immune system by promoting the production of antimicrobial peptides (AMPs) and intraepithelial lymphocytes (IELs). AMPs are small, positively charged proteins, secreted by Paneth cells in the epithelial membrane, that interact with and permeate bacterial membranes. IELs are regulatory CD8 T cells, found throughout the epithelial membrane, that also produce AMPs, such as alpha and beta defensins and cathelicidins. Furthermore, studies show that SCFAs, such as butyrate, can enhance IEL secretion of AMPs to further aid in antimicrobial protection [3]. In the intestines, extrarenal expression of 1-alpha hydroxylase in the intestinal epithelium converts biologically inactive Vitamin D into its active form. Vitamin D is then able to interact with VDR to stimulate AMP secretion by the epithelium [59]. These AMPs are harbored in the mucus layer produced by epithelial goblet cells. By residing in the mucus layer, they ensure that they can carry out their antimicrobial function against invading pathogens without harming the host microbiota.

As previously described, deficiency in Vitamin D and decreased Vitamin D/VDR signaling increases the risk of IEC apoptosis. A decrease in functioning IECs has a series of detrimental effects, including increased secretion of inflammatory markers and decreased secretion of AMPs. Regarding IELs, studies show that VDR-deficient mice have impaired CD8 T cell development, which also results in decreased AMP secretion [60]. Ultimately, impaired Vitamin D/VDR signaling hinders the antimicrobial function of the immune system in the intestinal epithelial layer.

When invading pathogens migrate past the epithelial layer and into the lamina propria, the innate immune system functions primarily through macrophage activity (Table 1). Macrophage activation begins with M2 (quiescent) macrophages identifying and responding to invading microbes [3]. This interaction triggers the macrophage to convert to the M1 phenotype, which activates the inflammasome pathway [61]. This pathway mediates the production of IL-1B and IL-18, as well as the recruitment of additional phagocytes, to clear the invading pathogens. Once these pathogens are cleared, the macrophage converts back to its M2 state and aids in tissue repair by secreting IL-10 and TGFB [3,61,62].

The process of macrophage activation is influenced by Vitamin D, whereby M2 macrophages increase the secretion of IL-10 and decrease the secretion of TNF-α. This occurs due to VDR binding to IKK kinase, which prevents the activation of NF-κB [18]. While we have already established that this same process suppresses IEC apoptosis through inhibition of PUMA, the prevention of NF-κB activation also provides this additional benefit through the activity of the innate immune system.

### 3.6. Adaptive Immune System

The adaptive immune system, unlike the innate immune system, is slower-acting and specific, requiring prior exposure to pathogens to mount an appropriate response [58]. This arm of the immune system has a memory component that allows for faster and stronger defense responses with repeated exposure to the same pathogen primarily through the activity of dendritic cells (DC), B cells, and T cells [3].

Dendritic cells (DCs) play a smaller, but notable, role in the adaptive immune system. These cells are responsible for identifying pathogens and presenting them to B cells and T cells to activate an immune response (Table 3) [63,64]. Vitamin D has been studied to promote the tolerogenic state, or immature state, of DCs by activating VDRs on these cells. Tolerogenic DCs, in contrast to mature DCs, play a significant role in immune surveillance, but not immune response. By inhibiting maturation of DCs and also promoting Treg cell differentiation, Vitamin D suppresses the potential for an overactive immune response and decreases the risk of inflammation from the excessive release of pro-inflammatory cytokines. Vitamin D also affects DCs by promoting immune tolerance. By maintaining a more tolerogenic state, DCs can more easily distinguish between harmful and harmless organisms, controlling over-activation and autoimmunity, which can provoke the development of some autoimmune conditions [64]. In addition, DCs, like T cells, can also produce various cytokines. Vitamin D affects DCs by inhibiting the production of these cytokines like interleukin 12 (IL-12), which induces the pro-inflammatory Th1 phenotype of CD4 cells, and promoting the secretion of anti-inflammatory IL-10 [64].

The primary mediators of the adaptive immune system are B cells and T cells (Table 3). B cells have been proven to be crucial to intestinal homeostasis and pathogen defense by promoting IgA-dominated immune responses [65]. Recent studies suggest that an IgG-dominant B cell response may be implicated in the pathogenesis of IBD, but further research in this area and on the effect of Vitamin D on B cells is needed [66]. In contrast, the relationship between T cells, Vitamin D, and bowel inflammation is much clearer. T cells (CD4 and CD8) are responsible for recognizing antigens and coordinating immune responses. The subsets of CD4 cells include Th1 cells (produce IFN-γ), Th2 cells (produce IL-4, IL-5, and IL-13), and Th17 cells (produce IL-17) [3]. These cytokines play a role in activating the signaling cascade that prompts B cells and other T cells to coordinate microbial defense.

Several studies have been conducted on the effect of Vitamin D on T cells. As previously established, VDRs are abundant throughout the immune system, including T cells, and can affect cell growth, activity, and differentiation. These studies have shown that Vitamin D suppresses differentiation of T cells to the Th1 cells through the inhibition of IL-12, the cytokine responsible for the development of this subset of T cells [67]. In addition, Vitamin D has also been shown to decrease differentiation into Th17 (pro-inflammatory) cells and increase the presence of T regulatory cells, which promote anti-inflammation [68]. These effects of Vitamin D on T cells result in a significant reduction in the production of pro-inflammatory cytokines and increased production of anti-inflammatory cytokines such as IL-10. It is important to note that studies have also revealed that after T cell activation, there is a delay in their response to Vitamin D, likely due to delays in VDR and Cyp27B_1_ upregulation. This delay allows for appropriate pathogen clearance of the T cell-mediated immune response. After pathogen clearance, Vitamin D affects T cell suppression to prevent an excessive and harmful immune response [69]. Molecular studies suggest that Vitamin D dis-equilibrium can lead to dysregulated T cell responses that can occur with improper activation of the adaptive immune system, which may trigger bowel inflammation and contribute to etiopathogenesis for IBD [70].

## 4. Conclusions

In conclusion, GI homeostasis is a complex process that is regulated by not only the GI system, but also the gut microbiome and the immune system. This equilibrium is maintained by several factors, one of which is Vitamin D. Through the signaling cascade initiated by Vitamin D/VDR complexes, Vitamin D has a profound influence in maintaining the GI epithelium, minimizing IEC inflammation and death, maintaining microbial diversity in the gut microbiome, and suppressing excessive inflammatory actions of the immune system. The effect that Vitamin D has on the intestinal epithelium, especially, through its influence on junctional proteins and IECs that form the epithelial barrier, highlights its potential role for preventative or therapeutic intervention against inflammatory conditions such as IBD. Additionally, the role Vitamin D plays in inhibiting pathways of unchecked cellular damage in the p53 pathways and through the immune system is integral in ensuring an appropriate immune response for protection from invasive pathogens without risking autoimmunity. Furthermore, Vitamin D has a significant effect in modifying the gut microbiota, especially in individuals with IBD. Vitamin D promotes the flourishment of beneficial bacteria and discourages the presence of bacteria that are commonly implicated in inflammatory conditions.

Given the growing body of evidence regarding Vitamin D deficiency and its connection to various inflammatory and autoimmune bowel diseases, it is reasonable to consider that maintaining adequate Vitamin D levels is beneficial in minimizing the progression and severity of these conditions [71]. In addition, further studies are warranted to elucidate the role of Vitamin D in GI homeostasis and determine if Vitamin D has potential to be a preventative or therapeutic intervention in conditions such as IBD, IMC, and other immune-mediated conditions.

## Figures and Tables

**Table 1:** Role of Vitamin D in gut homeostasis and in a pro-inflammatory state (potential role of Vitamin D in pro-inflammatory state is based on studies with animal models). Mucus layer: Animal models show Ca^2+^ to be essential to goblet cell production, with Vitamin D being a mediator of Ca^2+^ levels [3,12]. Epithelial layer: Vitamin D/DVR signaling in animal models results in promotion of tight junction formation, as well as PUMA activity inhibition, to prevent IEC apoptosis [13,14,15,16]. Lamina propria: Innate immune system activity in this layer decreases inflammatory cytokine release, a process enhanced by Vitamin D [14,17,18].

	Role of Vitamin D in Gut Homeostasis	Potential Role of Vitamin D Supplementation in a Pro-inflammatory State
Mucus Layer	- Maintains adequate mucus production of goblet cells	- Increase in mucus production by goblet cells to strengthen the mucus layer
Epithelium	- Promotes junctional protein production - Inhibits IEC apoptosis	- Inhibition of PUMA activity - Promotion of tight junction formation - Upregulation of Vitamin D/VDR may be preventative and/or therapeutic in IBD and IMC
Lamina Propria	- Regulates appropriate innate immune system response	- Vitamin D/VDR inhibits NF-κB activation, reducing the production of inflammatory cytokines

**Table 3:** Components of the adaptive immune system and their relationship to Vitamin D and intestinal inflammation [3,63,64,65,66,67,68,69,70].

	Role in Adaptive Immunity	Effects of Vitamin D	Implications for Inflammation and IBD
Dendritic Cells (DCs)	- Identify pathogens and present antigens to B and T cells to activate an immune response	- Promotes tolerogenic state of DCs - Suppresses Th1 (pro-inflammatory T cell phenotype) differentiation by inhibiting IL-12 - Increases IL-10 (anti-inflammatory cytokine) production - Increases anti-inflammatory Treg cell differentiation	- Reduces pro-inflammatory cytokine release - Reduces excessive immune activation and inflammation
B Cells	- Mediate pathogen defense by promoting IgA-dominant immune responses	- Role is unclear but possibility for immunoglobulin regulation	- IgA dominant immune responses promote intestinal homeostasis - May decrease IBD risk by shifting away from IgG-dominant immune responses
T Cells (CD4 and CD8)	- Recognize antigens and coordinate immune responses - CD4 subsets include: Th1 (produces IFN-γ) Th2 (produces IL-4, IL-5, and IL-13) Th17 (produces IL-17)	- Suppresses Th1 (pro-inflammatory T cell phenotype) differentiation by inhibiting IL-12 - Suppresses Th17 (pro-inflammatory T cell phenotype) differentiation - Increases IL-10 (anit-inflammatory cytokine) production - Increases anti-inflammatory Treg cell differentiation	- Reduces pro-inflammatory cytokine release - Reduces excessive immune activation and inflammation
